# Prognosis, imaging characteristics, and clinicopathological features of heavy smokers with clinical stage I lung adenocarcinoma: a multicenter study

**DOI:** 10.1007/s11748-025-02166-7

**Published:** 2025-06-10

**Authors:** Ikki Takada, Yoshihisa Shimada, Takahiro Mimae, Yujin Kudo, Takuya Nagashima, Yoshihiro Miyata, Hiroyuki Ito, Morihito Okada, Norihiko Ikeda

**Affiliations:** 1 Department of Surgery, Tokyo Medical University, 6-7-1 Nishi-Shinjuku, Shinjuku-ku, Tokyo, 161-0032 Japan; 2 Department of Thoracic Surgery, Kanagawa Cancer Center, 2-3-2 Nakao, Asahi-ward, Yokohama, Kanagawa 241-8515 Japan; 3 Department of Surgical Oncology, Hiroshima University, 1-2-3 Kasumi, Minami-ku, Hiroshima, Hiroshima 734-0037 Japan

**Keywords:** Heavy smoker, Adenocarcinoma, Prognostic factor, Smoking extent, SUVmax

## Abstract

**Objective:**

This study aimed to elucidate the relationship between smoking extent and prognosis, imaging characteristics, and clinicopathological factors in patients with clinical stage I lung adenocarcinoma (c-stage I LDA).

**Methods:**

We evaluated 2,285 patients who underwent surgical resection for c-stage I LDA between 2010 and 2018. Patients were classified into three groups based on the Brinkman Index (BI): never smokers (BI = 0), light smokers (0 < BI ≤ 600), and heavy smokers (BI > 600). Clinicopathological features and prognosis were analyzed according to smoking extent.

**Results:**

Significant differences in overall survival (OS) were observed across the smoking groups. Heavy smokers exhibited more invasive imaging characteristics, including a larger solid proportion and a higher maximum standardized uptake value (SUVmax), compared to never and light smokers. In multivariable analyses, heavy smoking was significantly associated with poorer OS (hazard ratio [HR] 2.071, *p* < 0.001). In addition, older age (HR 1.111, *p* < 0 .001) and the presence of vascular invasion (HR 2.312, *p* < 0.001) were also associated with worse OS among heavy smokers.

**Conclusion:**

Smoking extent was independently associated with poorer survival, larger solid tumor size, and higher SUVmax in patients with c-stage I LDA. Age and vascular invasion emerged as strong prognostic factors, particularly among heavy smokers.

## Introduction

Cigarette smoking is a well-established risk factor for lung cancer [1], which remains the leading cause of cancer-related mortality worldwide [2–4]. Numerous studies have demonstrated the prognostic impact of smoking status in patients with early-stage non-small cell lung cancer, regardless of histological type [5–7]. Cigarette smoking plays a critical role in the initiation and progression of lung adenocarcinoma (LDA), the most common histological subtype of lung cancer. Nevertheless, 10–40% of LDA cases occur in individuals with no history of smoking [8].

Despite these findings, relatively few studies have investigated the impact of smoking extent and continued smoking on survival and clinicopathological characteristics in smokers with LDA, or explored the underlying molecular mechanisms contributing to LDA tumorigenesis in smokers. Yoshino et al. reported that smoking status was a significant prognostic factor in patients with pathological stage I LDA, with a hazard ratio (HR) of 1.8 for overall survival (OS) compared to never smokers [9]. Similarly, Maeda et al. demonstrated that both smoking status and extent were significantly correlated with OS in patients with stage IA LDA, with heavy smokers showing a significantly worse prognosis [10].

Although these studies highlight that heavy smoking and greater smoking extent negatively affect prognosis in early-stage LDA, few have focused specifically on clinicopathological features and outcomes in heavy smokers with early-stage disease. Therefore, this multicenter study aimed to elucidate the relationship between smoking extent and prognosis, imaging characteristics, and clinicopathological factors in patients with clinical stage I LDA. In addition, it sought to identify specific prognostic factors for heavy smokers.

## Methods

### Study population

A total of 3,680 patients underwent complete surgical resection for lung cancer at three institutions (Tokyo Medical University, Hiroshima University, and Kanagawa Cancer Center) between January 2010 and December 2018. Complete resection was defined according to the criteria of the International Association for the Study of Lung Cancer Staging Committee [11].

All patients underwent preoperative computed tomography (CT) and fluorodeoxyglucose-positron emission tomography/CT (FDG-PET/CT) as part of routine lung cancer staging. Clinical staging was performed based on the 8th edition of the TNM classification. For cases diagnosed prior to 2017 under the 7th edition, clinical staging was retrospectively updated to align with the 8th edition criteria. Exclusion criteria included the following: (1) pathological diagnosis of small cell lung cancer, (2) large cell neuroendocrine carcinoma or carcinoid, (3) clinical stage 0 or II–IV disease, and (4) incomplete follow-up data. After applying these criteria, 2,285 patients with clinical stage I LDA were included in the analysis. Smoking status was collected from all patients preoperatively, including the Brinkman Index (BI), with 20 BI corresponding to smoking 20 cigarettes per day for 1 year. A BI of 600 was adopted as the cutoff to distinguish between light smokers (0 < BI ≤ 600) and heavy smokers (BI > 600), based on the eligibility criteria of the National Lung Screening Trial (NLST), in which participants had a smoking history of at least 600 BI. This retrospective study was approved by the institutional review boards of all participating centers, and the requirement for informed consent was waived.

### Radiological assessment of primary tumors

All patients in this study underwent preoperative CT and FDG-PET/CT. The whole tumor size, solid tumor size, and the consolidation-to-tumor ratio (CTR) defined as the ratio of the maximum diameter of consolidation to the maximum tumor diameter were measured. To minimize inter-institutional variation in standardized uptake values (SUVs), an anthropomorphic body phantom (NEMA NU2-2001, Data Spectrum Corp., Hillsborough, NC, USA) was used. A calibration factor was calculated by dividing the actual SUV by the measured mean SUV in the phantom background, thereby reducing variability in SUV measurements across institutions. The final SUV used in this study was referred to as the revised higher maximum SUV (SUVmax). The original SUVmax values were determined by radiologists at each participating institution.

### Pathological evaluations

All dissected lymph nodes were microscopically analyzed for metastatic infiltration. The size of the invasive component of the tumor was measured on pathological slides. Blood vessel invasion (V) and visceral pleural invasion (PL) were evaluated using hematoxylin and eosin (H&E) and Elastica van Gieson staining. Lymphatic vessel invasion (Ly) was evaluated using H&E staining and, when necessary, by lymphatic endothelial staining. If V, Ly, lymph node metastasis, or PL were identified, the patient was diagnosed as positive for pathological invasive factors. The histological subtypes of lung cancer were determined according to the World Health Organization classification. Pathological staging was conducted in line with the 7th edition of the TNM classification for lung cancer [12]. According to the grading criteria established by the International Association for the Study of Lung Cancer, LDAs are classified into three grades: well-differentiated (lepidic predominant, with < 20% high-grade patterns), moderately differentiated (acinar or papillary predominant, with < 20% high-grade patterns), and poorly differentiated (with ≥ 20% high-grade patterns) [13].

### Statistical analysis

OS was defined as the time from the date of surgery to the date of death from any cause or the last date the patient was confirmed to be alive. Recurrence-free survival (RFS) was defined as the time from the date of surgery to the date of first recurrence, death from any cause, or the last follow-up date. Survival curves for OS and RFS were generated using the Kaplan–Meier method and compared using the log-rank test. Univariable and multivariable analyses were performed to identify factors associated with unfavorable OS and RFS outcomes. Univariable analyses were performed using Pearson’s Chi-squared test for categorical variables and Student’s *t* test or one-way analysis of variance for continuous variables. Multivariable logistic regression models were developed using a backward stepwise selection method. The proportional hazards assumption was tested using the complementary log–log plot model. Variables with a *p* value < 0.15 in univariable analysis were considered candidates for inclusion in the stepwise selection procedure. All statistical tests were two-sided, and *p* values of < 0.05 were considered statistically significant. Analyses were performed using the SPSS statistical software (version 26.0; IBM Corp., Armonk, NY, USA).

## Results

Table 1 presents the patients’ characteristics. The median follow-up time for survivors was 35.0 months (range: 12.7–87.1 months). Among the patients, 1122 (49.1%), 459 (20.1%), and 704 (30.8%) were never, light, and heavy smokers, respectively. Compared with the other groups, heavy smokers were more likely to be male (heavy smokers vs never smokers, *p* < 0.001; heavy smokers vs light smokers, *p* < 0.001) and to have higher tumor SUVmax of tumor (heavy smokers vs never smokers, *p* < 0.001; heavy smokers vs light smokers, *p* = 0.002), poorly differentiated adenocarcinoma (heavy smokers vs never smokers, *p* < 0.001; heavy smokers vs light smokers, *p* < 0.001), epidermal growth factor receptor (EGFR) wild-type tumors (heavy smokers vs never smokers, *p* < 0.001; heavy smokers vs light smokers, *p* < 0.001), and local recurrence (heavy smokers vs never smokers, *p* < 0.001; heavy smokers vs light smokers, *p* < 0.001) (Table 1).

**Table 1:** Patient characteristics

Variables	Never smoker *N* = 1122 (%)	Light smoker *N* = 459 (%)	Heavy smoker *N* = 704 (%)	Never vs. light *p* value	Never vs. heavy *p* value	Light vs. heavy *p* value
Age, year, median (range)	67 (32–84)	63 (44–81)	67 (53–84)	< 0.001	0.310	< 0.001
Sex				< 0.001	< 0.001	< 0.001
Male	184 (16.4)	302 (65.8)	622 (88.4)			
Female	938 (83.6)	157 (34.2)	82 (11.6)			
Solid tumor size, cm, median, (range)	1.56 (0.36–4.01)	1.39 (0.56–3.50)	1.57 (0.07–3.04)	0.423	< 0.001	0.002
CTR				0.240	< 0.001	< 0.001
< 1	758 (67.6)	296 (64.5)	329 (46.7)			
1	364 (32.4)	163 (35.5)	375 (53.3)			
SUVmax of tumor, median (range)	2.01 (0.00–17.60)	1.40 (0.00–11.17)	2.52 (0.00–17.21)	0.001	< 0.001	0.002
Surgical procedure				0.085	0.122	0.005
Wedge resection	126 (11.2)	40 (8.7)	95 (13.5)			
Anatomical resection	996 (88.8)	419 (91.3)	609 (86.5)			
Subtype						
AIS	99 (8.8)	27 (5.9)	22 (3.1)			
MIA AAH	112 (10.0)	52 (11.3)	40 (5.7)			
Lepidic	306 (27.3)	112 (24.4)	126 (17.9)			
Papillary	336 (32.4)	163 (35.5)	281 (39.9)			
Ainar	157 (14.0)	56 (12.2)	99 (14.1)			
Solid	32 (2.9)	26 (5.7)	100 (14.2)			
Micropapillary	12 (1.1)	5 (1.1)	12 (1.7)			
Invasive mucinous	30 (2.7)	11 (2.4)	18 (2.6)			
Unknown	11 (1.0)	7 (1.5)	6 (0.9)			
Tumor differentiation				0.016	< 0.001	< 0.001
Well or moderate	1078 (96.1)	428 (93.2)	592 (84.1)			
Poor	44 (3.9)	31 (6.8)	112 (15.9)			
Lymphatic invasion				0.103	< 0.001	0.104
Negative	956 (85.2)	376 (81.9)	549 (78.0)			
Positive	166 (14.8)	83 (18.1)	155 (22.0)			
Vascular invasion				0.115	< 0.001	< 0.001
Negative	939 (83.7)	369 (80.4)	480 (68.2)			
Positive	183 (16.3)	90 (19.6)	224 (31.8)			
Pleural invasion				0.309	< 0.001	< 0.001
Negative	998 (88.9)	400 (87.1)	542 (77.0)			
Positive	124 (11.1)	59 (12.9)	162 (23.0)			
Pathological lymph node metastasis				0.499	0.015	0.013
Negative	1030 (91.8)	426 (92.8)	622 (88.4)			
Positive	92 (8.2)	33 (7.2)	82 (11.6)			
Adjuvant chemotherapy				0.784	0.064	0.230
Non or oral administration	1017 (90.6)	414 (90.2)	619 (87.9)			
Intravenous administration	105 (9.4)	45 (9.8)	85 (12.1)			
EGFR mutation				0.010	< 0.001	< 0.001
Negative	296 (26.4)	156 (34.0)	357 (50.7)			
Positive	519 (46.3)	192 (41.8)	129 (18.3)			
Unknown	307 (27.4)	111 (24.2)	218 (31.0)			
Postoperative recurrences				0.648	0.002	0.155
No recurrence	1038 (92.5)	423 (92.2)	630 (89.5)			
Local	13 (1.2)	8 (1.7)	25 (3.6)			
Distant/both local and distance	71 (6.3)	28 (6.1)	49 (7.0)			
Cause of death				0.115	< 0.001	< 0.001
Lung cancer deaths	19 (1.7)	15 (3.3)	26 (3.7)			
Deaths from other diseases	19 (1.7)	10 (2.2)	51 (7.2)			

*CTR* consolidation tumor ratio, *SUVmax* maximum standardized uptake value, *EGFR* epidermal growth factor receptor

**Fig. 1:**
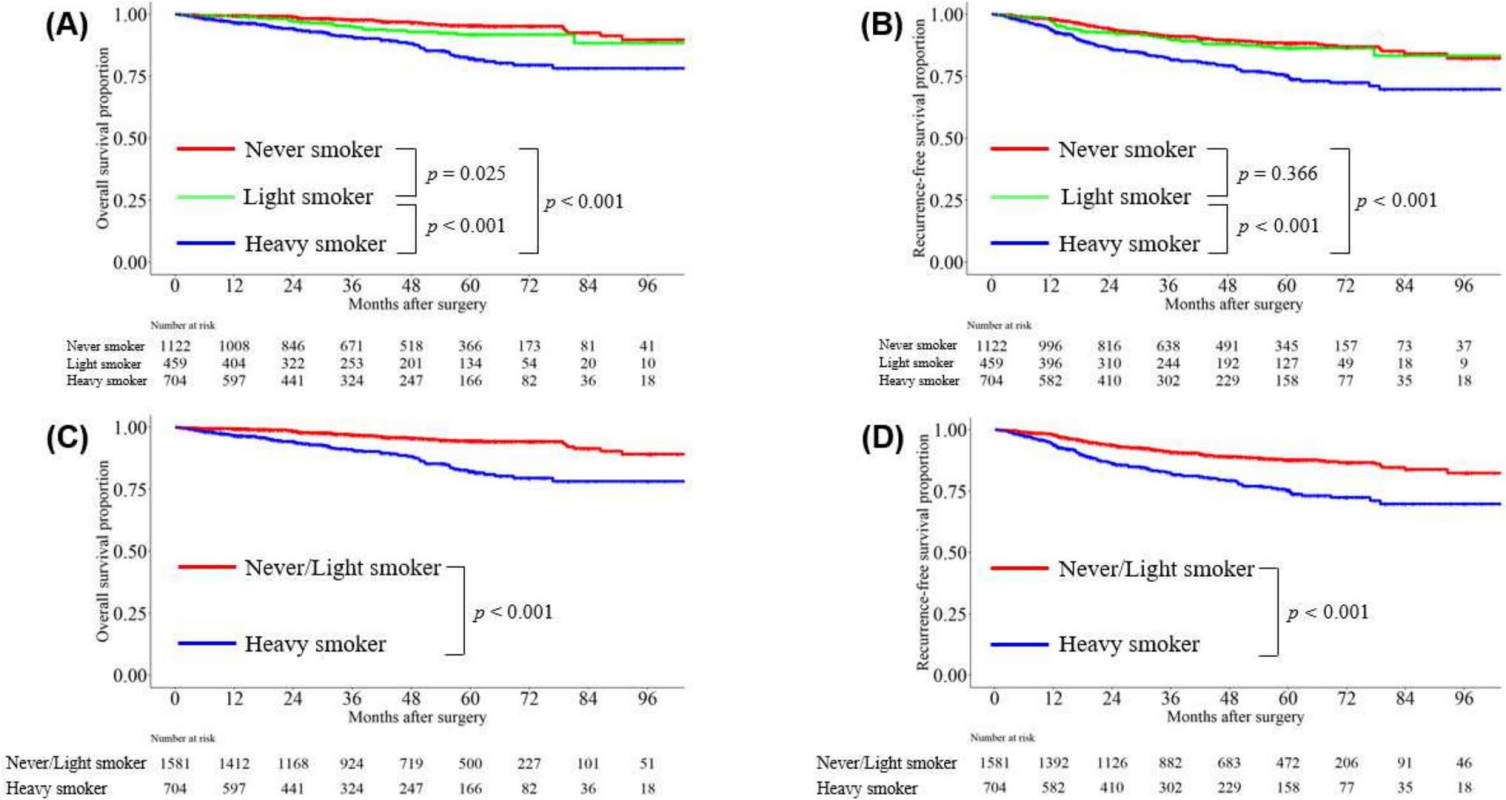
Overall survival (OS) and recurrence-free survival (RFS) among patients with clinical stage I lung adenocarcinoma according to smoking status. **A** OS and **B** RFS between the 3 groups. **C** OS and **D** RFS between heavy smokers and others

**Fig. 2:**
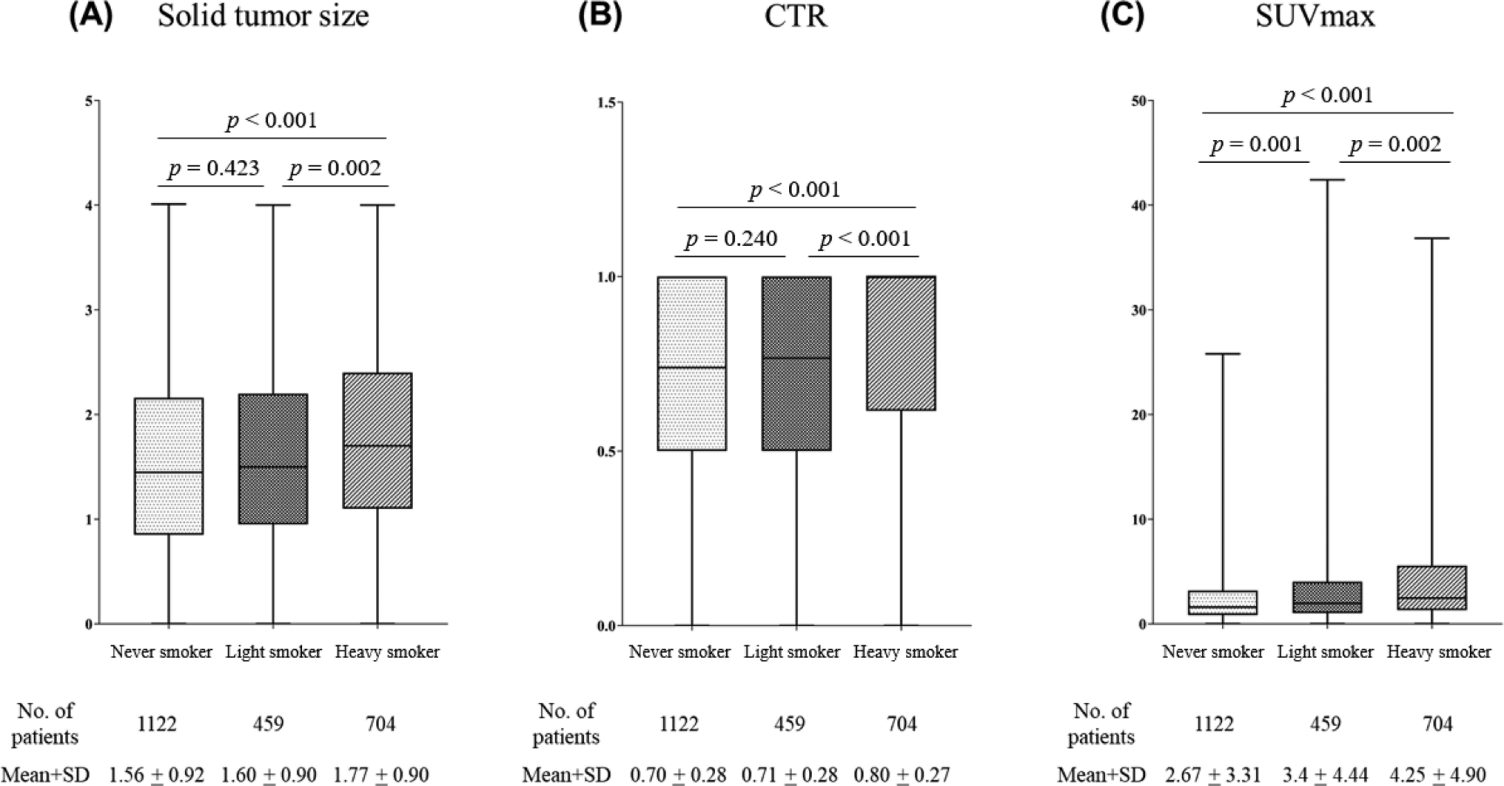
Box plots for the comparison of the distribution of **A** solid tumor size, **B** consolidation tumor ratio (CTR), and **C** maximum standardized uptake value (SUVmax) on F-18 fluorodeoxyglucose-positron emission tomography–computed tomography (FDG-PET/CT) to identify correlations by smoking history

Kaplan–Meier curves showed that the 5-year OS rates were 95.5, 91.8, and 82.2% for never, light, and heavy smokers, respectively (never smokers vs. light smokers, *p* = 0.025; never smokers vs. heavy smokers, *p* < 0.001; light smokers vs. heavy smokers, *p* < 0.001; Fig. 1A). Similarly, the 5-year RFS rates were 88.3, 86.3, and 75.3% for never smokers, light smokers, and heavy smokers, respectively (never smokers vs. light smokers, *p* = 0.366; never smokers vs. heavy smokers, *p* < 0.001; light smokers vs. heavy smokers, *p* < 0.001; Fig. 1B). When never and light smokers were grouped together and compared with heavy smokers, the combined group showed significantly better outcomes, with a 5-year OS rate of 94.5% and an RFS rate of 87.7% (never/light smokers vs. heavy smokers, *p* < 0.001 for both; Fig. 1C, D). To evaluate preoperative imaging findings based on smoking extent, we created box plots to visualize the distributions of solid-part size (heavy smokers vs never smokers, *p* < 0.001; heavy smokers vs light smokers, *p* = 0.002; Fig. 2A), CTR (heavy smokers vs never smokers, *p* < 0.001; heavy smokers vs light smokers, *p* < 0.001; Fig. 2B), and tumor SUV max (heavy smokers vs never smokers, *p* < 0.001; heavy smokers vs light smokers, *p* = 0.002; Fig. 2C). Statistically significant differences in all three parameters were observed between heavy smokers and never/light smokers.

Multivariable analysis revealed that older age (hazard ratio [HR] 1.085, *p* < 0.001), male sex (HR 0.555, *p* = 0.010), heavy smoking (HR 2.071, *p* < 0.001), poorly differentiated adenocarcinoma (HR 1.616, *p* = 0.032), vascular invasion (HR 1.937, *p* = 0.002), and pathological lymph node metastasis (HR 1.705, *p* = 0.031) were independently associated with poor OS (Table 2). Heavy smokers were identified as a distinct subgroup with poor prognosis and more aggressive tumor feature. Therefore, univariable and multivariable analyses were performed exclusively for the 704 heavy smokers (Table 3). In univariable analysis, age (HR 4.949, *p* < 0.001), male sex (HR 0.193, *p* = 0.022), CTR (HR 1.712, *p* = 0.022), tumor SUVmax (HR 1.053, *p* = 0.001), tumor differentiation (HR 1.927, *p* = 0.013), lymphatic invasion (HR 2.097, *p* = 0.002), vascular invasion (HR 2.284, *p* < 0.001), pleural invasion (HR 1.653, *p* = 0.037), and pathological lymph node metastasis (HR 2.079, *p* = 0.013) were found to be prognostic for OS. Multivariable analysis showed that older age (HR 1.111, *p* < 0.001) and vascular invasion (HR 2.312, *p* < 0.001) were independent prognostic factors for OS in the subgroup (Table 3).

**Table 2:** Multivariable analysis for overall survival in all patients

Variables	Multivariable analysis	
	HR (95% CI)	*p* value
Age	1.085 (1.062–1.109)	< 0.001
Sex	0.555 (0.354–0.869)	0.010
Heavy smoker	2.071 (1.376–3.117)	< 0.001
Poorly differentiate	1.616 (1.042–2.506)	0.032
Vascular invasion	1.937 (1.272–2.949)	0.002
Lymph node metastasis	1.705 (1.050–2.767)	0.031

*HR* hazard ratio, *CI* confidence interval, *CTR* consolidation tumor ratio, *SUV* standardized uptake value

**Table 3:** Univariable and multivariable analysis for overall survival in heavy smoker

Variables	Univariable analysis		Multivariable analysis	
	HR (95% CI)	*p* value	HR (95% CI)	*p* value
Age	4.949 (2.762–8.867)	< 0.001	1.111 (1.077–1.147)	< 0.001
Sex	0.193 (0.047–0.786)	0.022		
Solid tumor size	1.255 (0.987–1.595)	0.064		
CTR = 1 or not	1.712 (1.082–2.710)	0.022		
SUVmax of tumor	1.053 (1.020–1.086)	0.001		
Surgical procedure	0.835 (0.524–1.328)	0.445		
Differentiation	1.927 (1.147–3.235)	0.013		
Lymphatic invasion	2.097 (1.313–3.350)	0.002		
Vascular invasion	2.284 (1.461–3.572)	< 0.001	2.312 (1.434–3.726)	< 0.001
Pleural invasion	1.653 (1.030–2.652)	0.037		
Pathological lymph node metastasis	2.079 (1.165–3.713)	0.013		
Adjuvant chemotherapy	0.603 (0.289–1.257)	0.177		

*HR* hazard ratio, *CI* confidence interval, *CTR* consolidation tumor ratio, *SUV* standardized uptake value

## Discussion

The present study yielded several important findings. First, heavy smokers had clinically and pathologically more aggressive malignant LDAs compared to never and light smokers. Second, the OS and RFS of heavy smokers were significantly worse than those of never and light smokers. Third, heavy smokers exhibited more invasive imaging characteristics, with a larger solid proportion on CT and a higher SUVmax. Finally, age and the presence of vascular invasion were identified as independent prognostic factors for OS among heavy smokers.

Cigarette smoking is associated with the development of various histological types of lung cancer, particularly squamous cell and small cell carcinomas. Previous studies indicated that both smoking history and smoking extent were also poor prognostic factors in patients with LDA [3, 14–17]. Yoshino et al. [9] reported that never and light smokers had a more favorable prognosis compared to heavy smokers. Maeda et al. [10, 18] found that smoking extent was involved in OS in patients with pathological stage IA LDA. They showed that patients with a smoking history of > 600 BI had significantly more moderately or poorly differentiated carcinomas and a higher frequency of intratumoral vascular invasion than those with 600 or less BI, which is consistent with the findings of the present study. Shima et al. [19] reported that the incidence of pathological vascular invasion was significantly associated with the preoperative smoking extent in patients with stage I LDA, suggesting that cigarette smoking may contribute to the promotion of the metastatic process even in early-stage LDAs. Furthermore, a large prospective cohort study showed that, among heavy smokers, the risk of developing LDA was higher than that of developing squamous cell or small cell carcinoma, compared to never smokers [20]. These studies suggest that tobacco smoke exposure plays a more significant role in the initiation and progression of LDA biology more than previously recognized.

Several studies have suggested that chronic tobacco use is associated with increased glucose metabolic activity, which may cause baseline elevation of the SUVmax in the lungs of smokers. Vanfleteren et al. [20] reported that patients with chronic obstructive pulmonary disease (COPD) had a significantly increased FDG uptake in visceral adipose tissue compared with non-COPD controls. A plausible explanation is that nicotine causes the greatest increase in FDG uptake in adipose tissue compared to other interventions [21]. In the present study, smoking status was found to be associated with the glucose uptake activity of lung tumors. A high SUV of primary tumors was reported to be significantly associated with occult N2 metastasis in never smokers but not in smokers [22]. Maoqing et al. [23] demonstrated that the SUVmax of lung tumors was significantly higher in smokers than in never smokers, which was prominently shown in patients with an increased cumulative smoking dose.

These results indicate the clinical usefulness of the glucose activity data of primary tumors that differ according to smoking history and extent. Smoking extent is an adverse prognostic factor after surgical resection of lung cancer, but few studies have focused on identifying prognostic factors exclusively in heavy smokers with lung cancer. The present study demonstrated that age and the presence of vascular invasion were independent prognostic factors; however, the reason for the extraction of these two factors from many clinicopathological risk factors is not clear. Cigarette smoking affects evolution, pathological features, molecular characteristics, the efficacy of treatment, and overall outcomes. In elderly patients, a long-term smoking history is the cause of not only cancer but also heart disease, stroke, and other lung diseases. Moreover, elderly heavy smokers with various comorbidities have a lower chance of receiving platinum-based adjuvant chemotherapies and aggressive post-recurrent systemic therapies, such as immune checkpoint inhibitors plus chemotherapies, than young heavy smokers. Therefore, it is plausible that older adults who smoke heavily have poorer health status due to their frailty and various smoking-related diseases than light or never smokers, likely resulting in an extremely poor prognosis. Vascular invasion is a fundamental step in hematogenous and lymphatic metastases. The pathophysiological effects of nicotine are mediated by nicotinic acetylcholine receptors, which are expressed in a variety of cells including endothelial cells and several histological types of lung tissue. Nicotine induces angiogenesis by upregulating many molecules, such as COX-2, prostacyclin, and vascular endothelial growth factor (VEGF). Nicotine can also exert its pro-angiogenic activity by increasing cellular levels of VEGF, basic fibroblast growth factor, and platelet-derived growth factor. Thus, multiple molecules are involved in carcinogenesis and invasion, and the mechanisms induced by the accumulation of nicotine may facilitate a more aggressive form of vascular invasion and angiogenesis as pro-metastatic events in heavy smokers with lung cancer.

In the present study, “smoking” referred exclusively to conventional cigarette smoking and did not include secondhand smoke, electronic cigarette smoke (ESC) or vaping. However, Tang et al. reported that ESC exposure in mice induced lung cancer and bladder urothelial hyperplasia. ECS was shown to generate γ-OH-PdG and O^6^-methyl-dG adducts in the lungs and bladder urothelium, while also inhibiting DNA repair mechanisms in lung tissues. ECS devices and vaping fluids have been found to contain several known and probable carcinogens, including nicotine derivatives, polycyclic aromatic hydrocarbons, heavy metals, aldehydes, and other complex organic compounds [25]. Although the carcinogenicity of ECS has been demonstrated in animal models, evidence in humans remains limited and is primarily based on epidemiological studies. Given that the rapid rise in ECS use over the past decade, it is essential to evaluate the carcinogenic potential and clinical impact of ECS and vaping exposure, particularly in cohorts undergoing lung cancer surgery.

This study had several limitations. First, the multicenter design suggests a possibility of inter-center differences in the clinical staging by CT imaging and pathological diagnostic criteria. Second, information on smoking status and extent was reported by patients but was not confirmed biochemically. Third, despite the use of an anthropomorphic phantom to minimize differences, inter-institutional variability may be present in the SUVs obtained. Fourth, the data in this study did not reflect the effects of secondhand smoke.

In conclusion, chronic cigarette smoking has different effects on the long-term survival of patients and the pathological malignancy grades of lung tumors. Since LDAs arising in heavy smokers are metabolically more active and has a higher malignancy potential than those in light and never smokers, our findings support asymptomatic heavy smokers receiving an active screening program for early detection of lung cancer. Aggressive postoperative adjuvant therapies can be considered for heavy smokers with vascular invasion-positive LDA owing to their unfavorable prognosis, even in the absence of lymph node metastasis. 

## Data Availability

The data related to this study are available from the corresponding author upon reasonable request.
